# Deletion of VPS50 protein in mouse brain impairs synaptic function and behavior

**DOI:** 10.1186/s12915-024-01940-y

**Published:** 2024-06-26

**Authors:** Constanza Ahumada-Marchant, Carlos Ancatén-Gonzalez, Henny Haensgen, Bastian Brauer, Nicolas Merino-Veliz, Rita Droste, Felipe Arancibia, H. Robert Horvitz, Martha Constantine-Paton, Gloria Arriagada, Andrés E. Chávez, Fernando J. Bustos

**Affiliations:** 1 Constantine-Paton Research Laboratory, Institute of Biomedical Sciences (ICB), Faculty of Medicine and Faculty of Life Sciences, Universidad Andres Bello, Santiago, Chile; 2 Department of Biology, Howard Hughes Medical Institute, Massachusetts Institute of Technology, McGovern Institute for Brain Research, Cambridge, MA 02139 USA; 3 Department of Biology, Massachusetts Institute of Technology, McGovern Institute for Brain Research, Cambridge, MA 02139 USA; 4 Programa de Doctorado en Ciencias, Universidad de Valparaíso, Mención Neurociencia, Valparaíso, Chile; 5 Instituto de Neurociencias, Centro Interdisciplinario de Neurociencia de Valparaíso (CINV), Facultad de Ciencias, Universidad de Valparaíso, Valparaíso, Chile; 6 Millennium Nucleus of Neuroepigenetics and Plasticity (EpiNeuro), Santiago, Chile

**Keywords:** ASD, VPS50, Synaptic vesicles, Gene editing, Vesicle acidification

## Abstract

**Background:**

The VPS50 protein functions in synaptic and dense core vesicle acidification, and perturbations of VPS50 function produce behavioral changes in *Caenorhabditis elegans*. Patients with mutations in VPS50 show severe developmental delay and intellectual disability, characteristics that have been associated with autism spectrum disorders (ASDs). The mechanisms that link VPS50 mutations to ASD are unknown.

**Results:**

To examine the role of VPS50 in mammalian brain function and behavior, we used the CRISPR/Cas9 system to generate knockouts of VPS50 in both cultured murine cortical neurons and living mice. In cultured neurons, KO of VPS50 did not affect the number of synaptic vesicles but did cause mislocalization of the V-ATPase V1 domain pump and impaired synaptic activity, likely as a consequence of defects in vesicle acidification and vesicle content. In mice, mosaic KO of VPS50 in the hippocampus altered synaptic transmission and plasticity and generated robust cognitive impairments.

**Conclusions:**

We propose that VPS50 functions as an accessory protein to aid the recruitment of the V-ATPase V1 domain to synaptic vesicles and in that way plays a crucial role in controlling synaptic vesicle acidification. Understanding the mechanisms controlling behaviors and synaptic function in ASD-associated mutations is pivotal for the development of targeted interventions, which may open new avenues for therapeutic strategies aimed at ASD and related conditions.

**Supplementary Information:**

The online version contains supplementary material available at 10.1186/s12915-024-01940-y.

## Background

Thousands of mutations have been implicated in promoting neurodevelopmental disorders, with different degrees of certainty and a myriad of phenotypes [1–6]. An understanding of the consequences of specific gene mutations will be crucial to identifying common pathways and potential therapies for these disorders. Autism spectrum disorders (ASDs) are neurodevelopmental disorders characterized by deficits in social interaction, repetitive behaviors, and anxiety [7]. In addition, memory performance involving working and spatial memory is severely reduced in ASD patients [8, 9]. ASD can be driven by mutations that cause changes in brain wiring, structure, and function. Mutations in more than 1300 genes—including single-nucleotide polymorphisms (SNPs), copy-number variations (CNVs), deletions, and duplications—are associated with ASD, reflecting the complexity of its multifactorial genetics [1–6]. In particular, mutations that affect the function, loading, or availability of synaptic vesicles have been strongly associated with the manifestation of ASD-like phenotypes [10]. For instance, mice carrying mutations in Syn1, a synaptic vesicle component, cause limited synaptic vesicle release and, in Nhe9, a Na + /H + exchanger, cause hyper-acidification of vesicles; both mice mutants show phenotypes associated with ASD [11–13].

The evolutionarily conserved VPS50 protein functions in synaptic and dense-core vesicle maturation and acidification both in *Caenorhabditis elegans* and in murine cultured neurons and controls behavioral state in *C. elegans;* VPS50 is widely expressed in both the *C. elegans* and the murine nervous system [14]. VPS50 is associated with components of the Golgi-associated retrograde protein (GARP) complex and more specifically with the endosome-associated recycling protein (EARP) complex, suggesting a role in endocytosis of synaptic vesicles by early endosomes [15, 16]. In *C. elegans* VPS50 physically interacts with the protein VHA-15, a component of the V-ATPase complex pump responsible for acidifying both dense core and synaptic vesicles, and knockdown of VPS50 in cultured murine neurons causes a robust decrease in synaptic vesicle acidification [14]. In humans, a deletion spanning the *Vps50* gene, and the calcitonin receptor, has been reported in an ASD patient [17]. More recently, two patients with a neurodevelopmental disorder including severe developmental delays and intellectual disability were reported to carry homozygous loss-of-function mutations in *Vps50* [18]. These findings implicate VPS50 in ASD. However, no mechanisms have been identified that link the phenotypes shown by these patients to their deficits in *Vps50* function.

Below we describe our use of the CRISPR/Cas9 system to produce VPS50 knockouts (KO) in cultured murine neurons and mosaic knockouts (mKOs) in living mice, allowing us to analyze the role of VPS50 in synaptic function and mouse behavior. Our findings suggest mechanisms through which VPS50 mutations contribute to ASD phenotypes in general and to cognitive impairment in particular. In addition, our brain mosaic VPS50 mKO animal model should enable further investigations into the cellular and molecular mechanisms underlying the function of VPS50 and its relationship to ASD.

## Results

### VPS50 modulates synaptic vesicle acidification and V-ATPase V1 domain pump localization in cortical neurons

In *C. elegans*, VPS50 KO causes changes in behavior state, likely as a consequence of defects in synaptic and dense core vesicle acidification [14]. In mice, VPS50 is highly expressed in the central nervous system, and its knock-down in culture causes dysregulation of synaptic vesicle acidification [14]. To further investigate the role of VPS50 in regulating mammalian synaptic function and behavior, we used the CRISPR/Cas9 technology to generate VPS50 KO murine neurons. Cortical neuron cultures from Cas9 KI mice were transduced at 3 DIV with AAV encoding tdTomato as an infection marker alone or together with sgRNAs targeting the *Vps50* gene. The transduction efficiency reached > 90% of cultured neurons. Genomic DNA was extracted 10 days after infection, and a T7 endonuclease I assay was performed to determine the efficiency of gene editing. We tested different combinations of sgRNAs targeting the *Vps50* genomic locus and observed the highest editing efficiency when the pair sgRNA1 and sgRNA6 were used together (Additional file 1: Fig. S1A).

**Fig. 1:**
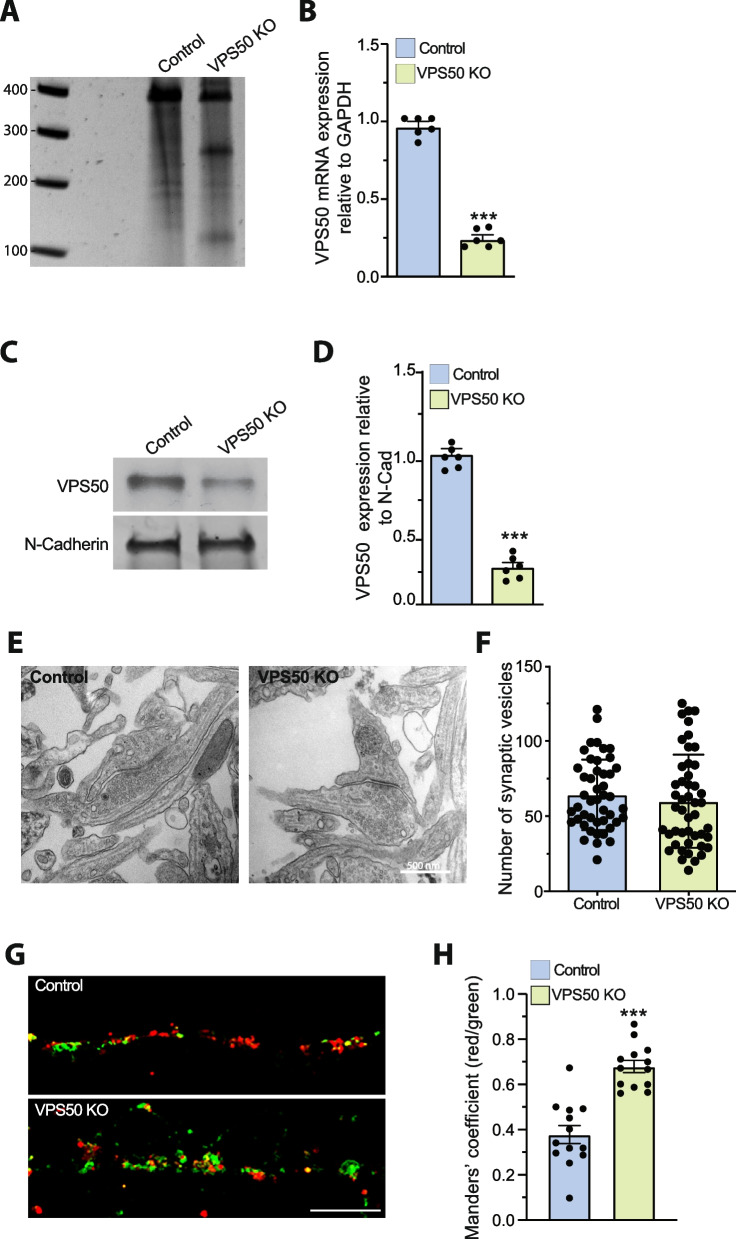
VPS50 gene editing causes a decrease in synaptic vesicle acidification but no change in synaptic vesicle number. Cortical neurons from Cas9KI mice were transduced with sgRNA targeting VPS50 at 3 DIV to produce VPS50 KO. **A** T7 endonuclease I assay of control or VPS50 KO cortical neurons. **B** RT-qPCR to quantify relative mRNA expression of VPS50. **C** VPS50 protein expression in VPS50 KO and control neurons. N-cadherin was used as loading control. **D** Quantification of VPS50 expression in western blots (*n* = 6 biological replicates). **E**, **F** Representative images of synaptic terminals as visualized by electron microscopy and quantification of the number of synaptic vesicles in control and VPS50 KO neurons (*n* = 50 cells per condition from 3 independent experiments). **G** Representative images of the Ratio-SyPhy signal used to determine synaptic vesicle acidification in control and VPS50 mKO neurons. Red signal shows all synaptic vesicles and green signal shows vesicles with basic pH. **H** Quantification of Ratio-SyPhy signal showing Mander’s coefficient. Thirteen samples from 3 biological replicates were used for Ratio-SyPhy quantification. Scale bar: 400 nm (**E**); 25 μm (**G**). Unpaired *t*-test was used for statistical analysis; ***p < 0.001. Error bars represent ± SEM. ***p < 0.001

Next, we performed a series of experiments using together sgRNA1 and sgRNA6 (hereafter referred to as “VPS50 KO” in culture and “VPS50 mKO” in mice). T7 endonuclease I assay revealed that in cultured neurons VPS50 KO allows efficient gene editing (Fig. 1A). Editing of the *Vps50* locus caused a ~ 70% reduction in VPS50 mRNA and protein levels in cortical neurons (Fig. 1B–D), confirming an efficient KO of VPS50 after gene editing. Locus-specific sequencing showed the resulting edited genomic sequences that were introduced by CRISPR/Cas9 as evidenced by the presence of deletions represented by dashes when compared to the WT sequence (Additional file 1: Fig. S1B). Since VPS50 has been related to synaptic vesicle acidification, we next evaluated if the number of vesicles was changed in VPS50 KO neurons. Importantly, VPS50 KO neurons showed no significant difference in the total number of synaptic vesicles as assessed by electron microscopy (Fig. 1E, F). To determine changes in synaptic vesicle acidification, we used the ratio-SyPhy probe, which in red signal shows all synaptic vesicles and in green the vesicles with basic pH, we observed a robust reduction in vesicle acidification in VPS50 KO neurons (Fig. 1G). Quantification using a Mander’s coefficient to show the ratio of red clusters (vesicles) that are also green (high pH) shows a reduction in the coefficient (Fig. 1H), consistent with previous observations showing that VPS50 modulates synaptic vesicle acidification [14].

**Fig. 2:**
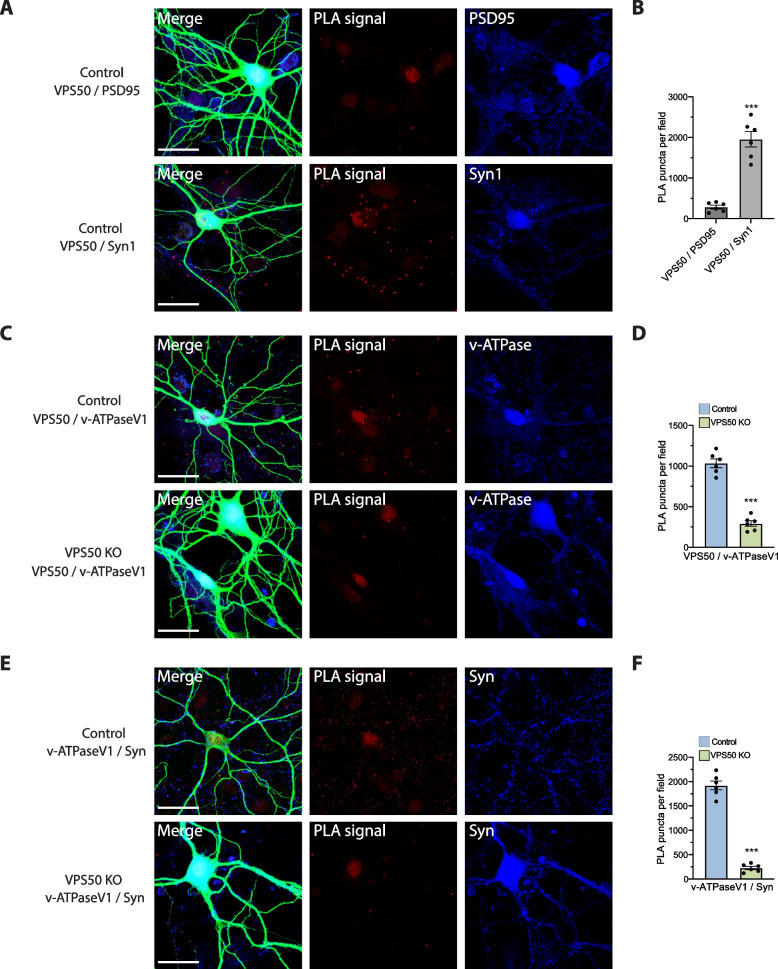
VPS50 KO causes mislocalization of v-ATPaseV1 domain in cortical neurons. Proximity-ligation assays (PLAs) was used to determine proximity between studied proteins as indicated in each case. A positive PLA signal, thus proximity between the proteins, is observed as red puncta in images (red channel, 546 nm). In all experiments, an antibody against the microtubule-associated protein 2 (MAP2) was used to stain processes of all neurons (green channel, 488 nm). Merge shows superimposed images of MAP2 (green), PLA signals (red), and the signal for a protein of interest as shown in the figure (blue, 633 nm). **A** PLA using PSD95 or Synapsin1 (Syn1) antibodies to define pre- or post-synaptic localization of VPS50 in control neurons. **B** Quantification of PLA puncta per field of VPS50/PSD95 and VPS50/Syn1. **C** PLA to assess proximity of VPS50 and v-ATPAseV1 domain in control and VPS50 KO neurons. **D** Quantification of PLA puncta per field of VPS50/v-ATPaseV1 in control and VPS50 KO neurons. **E** PLA to determine proximity of v-ATPaseV1 domain and Synaptophysin (Syn) in control and VPS50 KO neurons. **F** Quantification of PLA puncta per field of v-ATPaseV1 domain/Syn in Control and VPS50 KO neurons. Six low magnification (20 ×) fields from 3 independent biological samples were used for quantification in each condition. Scale bars, 25 μm. Unpaired *t*-test was used for statistical analysis; ***p < 0.001. Error bars represent ± SEM

As VPS50 is enriched in synaptic and dense core vesicles and appears as a soluble protein in mouse brain extracts [14], we next performed proximity ligation assays (PLA) to evaluate its approximate location within the synapse. PLA assesses the proximity between two proteins of interest by generating a positive fluorescent signal. In this study, the positive signal is observed as puncta in the red channel (550 nm), indicating that the targeted proteins are within a distance of < 40 nm. First, we tested in control neurons whether VPS50 was proximal to either the post-synaptic protein PSD95 or the presynaptic protein in synaptic vesicles Synapsin1. We observed the PLA signal only in proximity to Synapsin1, confirming that VPS50 is near or within synaptic vesicles (Fig. 2A). This interaction was quantified showing high PLA puncta only in the VPS50/Syn1 condition (Fig. 2B). As controls, we performed PLA reactions using Synapsin1/Synaptophysin and PSD95/Synaptophysin (Additional file 1: Fig. S2A-B). Since VPS50 interacts directly with ATP6V1H in murine tissue [14] and synaptic vesicle acidification is reduced in VPS50 KO neurons [14] (Fig. 1G, H), it is possible that VPS50 facilitates the sorting or assembly of the V-ATPase complex in synaptic vesicles to acidify them for neurotransmitter filling. To test this possibility, we used PLA to evaluate the proximity of VPS50 and the V-ATPaseV1 domain of the pump in control neurons and observed PLA signal, indicating proximity between VPS50 and the V-ATPaseV1 domain (Fig. 2C; top). Importantly, in VPS50 KO neurons, this PLA signal was absent, confirming the reduced expression of VPS50 and a consequent lack of interaction between VPS50 and the V-ATPaseV1 domain of the pump (Fig. 2C; bottom). Quantification of the PLA signal between VPS50/v-ATPaseV1 shows a significant decrease in the number of puncta per field in VPS50 KO neurons (Fig. 2D). We then asked if the localization of the V-ATPaseV1 domain is disrupted in VPS50 KO neurons. While PLA experiments examining the V-ATPaseV1 domain and Synaptophysin showed that they are in proximity in control neurons (Fig. 2E; top), no corresponding signal was observed in VPS50 KO neurons (Fig. 2E; bottom), strongly suggesting that KO of VPS50 causes a mislocalization of the V-ATPaseV1 domain. Quantification of PLA signal between v-ATPaseV1/Syn shows a significant decrease in VPS50 KO neurons (Fig. 2F). As a control to show that there are no changes in the expression of the interacting proteins, in all PLA experiments, we imaged in the blue channel (633 nm) the proteins of interest that were used to test interaction (Fig. 2). As an additional control for PLA experiments, we carried out western blot analyses of PSD95 and Synaptophysin in VPS50 KO neurons. As expected, we found a small reduction in PSD95 expression and no change in Synaptophysin expression levels (Additional file 1: Fig. S2I). Together, these results indicate that KO of VPS50 does not affect the total number of synaptic vesicles but produces a mislocalization of the V-ATPaseV1 domain that could in turn prevent the correct assembly of the v-ATPase complex and as a consequence could be likely responsible for the observed impairment in synaptic vesicle acidification [14] (Fig. 1G, H).

### VPS50 KO impairs synaptic activity in cortical neurons

**Fig. 3:**
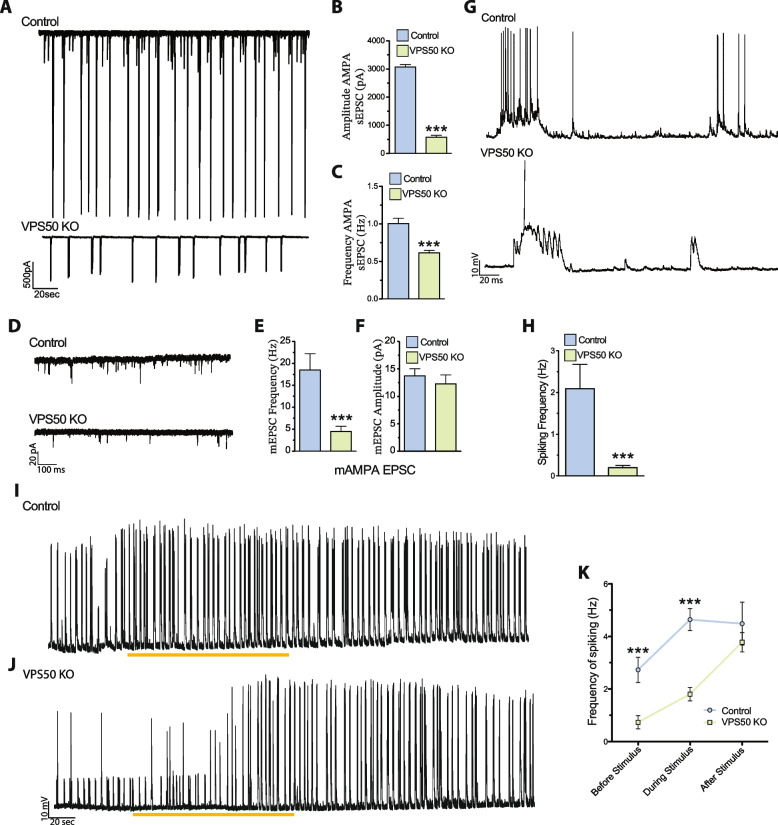
VPS50 KO neurons show a reduction in synaptic activity that can be recovered by inducing synaptic vesicle acidification. Cortical neuronal cultures from Cas9KI animals were transduced at 3DIV and at 12–13 DIV used for electrophysiology or calcium imaging. **A** Representative traces and **B**, **C** quantification of amplitude (**B**) and frequency (**C**) of spontaneous AMPA-mediated EPSCs. **D** Representative traces and **E**, **F** quantification of the frequency (**E**) and amplitude (**F**) of miniature AMPA-mediated currents. **G** Representative traces and quantification of the frequency (**H**) of current clamp recordings of control and VPS50 KO neurons. **I**–**K** Representative traces of current clamp recordings of control and VPS50 KO neurons expressing pHoenix. Orange bars under the traces show the periods pHoenix was activated. **K** Quantification of the frequency of spiking of control and VPS50 KO neurons before, during, and after stimulus with pHoenix to artificially acidify synaptic vesicles. For all experiments, at least 18 neurons from 3 independent experiments were analyzed. Unpaired *t*-test was used for statistical analysis; ***p < 0.001. Error bars represent mean ± SEM

To further evaluate the effect of VPS50 KO on synaptic function, murine cortical neurons were infected at 3 DIV and miniature and spontaneous excitatory postsynaptic currents (mEPSC and sEPSC, respectively) were recorded at 12–13 DIV. Compared to control neurons, VPS50 KO neurons showed a strong reduction in both the amplitude and frequency of sEPCS (Fig. 3A–C). To determine if the amplitude and frequency of sEPSC decreased with time, we performed continuous sEPSC recordings for 30 min. While in control neurons we observed no significant changes, VPS50 KO neurons displayed a significant reduction in the amplitude of the sEPSCs over time (Additional file 1: Fig. S3A-C), consistent with the idea that vesicular content might be reduced in VPS50 KO neurons. Moreover, VPS50 KO neurons showed a drastic reduction in the frequency but not in the amplitude of mEPSCs (Fig. 3D–F), an effect that could reflect an increase in the exocytosis of empty vesicles. This reduction in synaptic strength correlates with a decrease in neuronal activity, as the spontaneous spike frequency was decreased in VPS50 KO neurons compared to control neurons (Fig. 3G, H). In addition, calcium imaging using the genetically encoded GCaMP7 probe showed that VPS50 KO neurons were reduced in calcium event frequency, confirming deficient spiking (Additional file 1: Fig. S3D-E). To further evaluate if synaptic vesicle acidification was partly responsible for the reduction in vesicle content and neuronal activity, we used pHoenix, a genetically encoded proton pump targeted to synaptic vesicles [19]. Spontaneous spiking of pHoenix-infected control and VPS50 KO neurons was recorded for 1 min as baseline, and then, 532 nm light was used to activate pHoenix (yellow line under traces). During the 2-min activation period, we observed a partial recovery of spiking in VPS50 KO neurons close to control levels (Fig. 3I, J; yellow line). Moreover, after stimulation, we observed no significant differences from control neurons (Fig. 3K). Altogether, our results strongly indicate that the reduction in synaptic activity in VPS50 KO requires synaptic vesicle acidification, since this reduction can be rescued by artificially acidifying synaptic vesicles.

### VPS50 mKO impacts synaptic function and memory formation

**Fig. 4:**
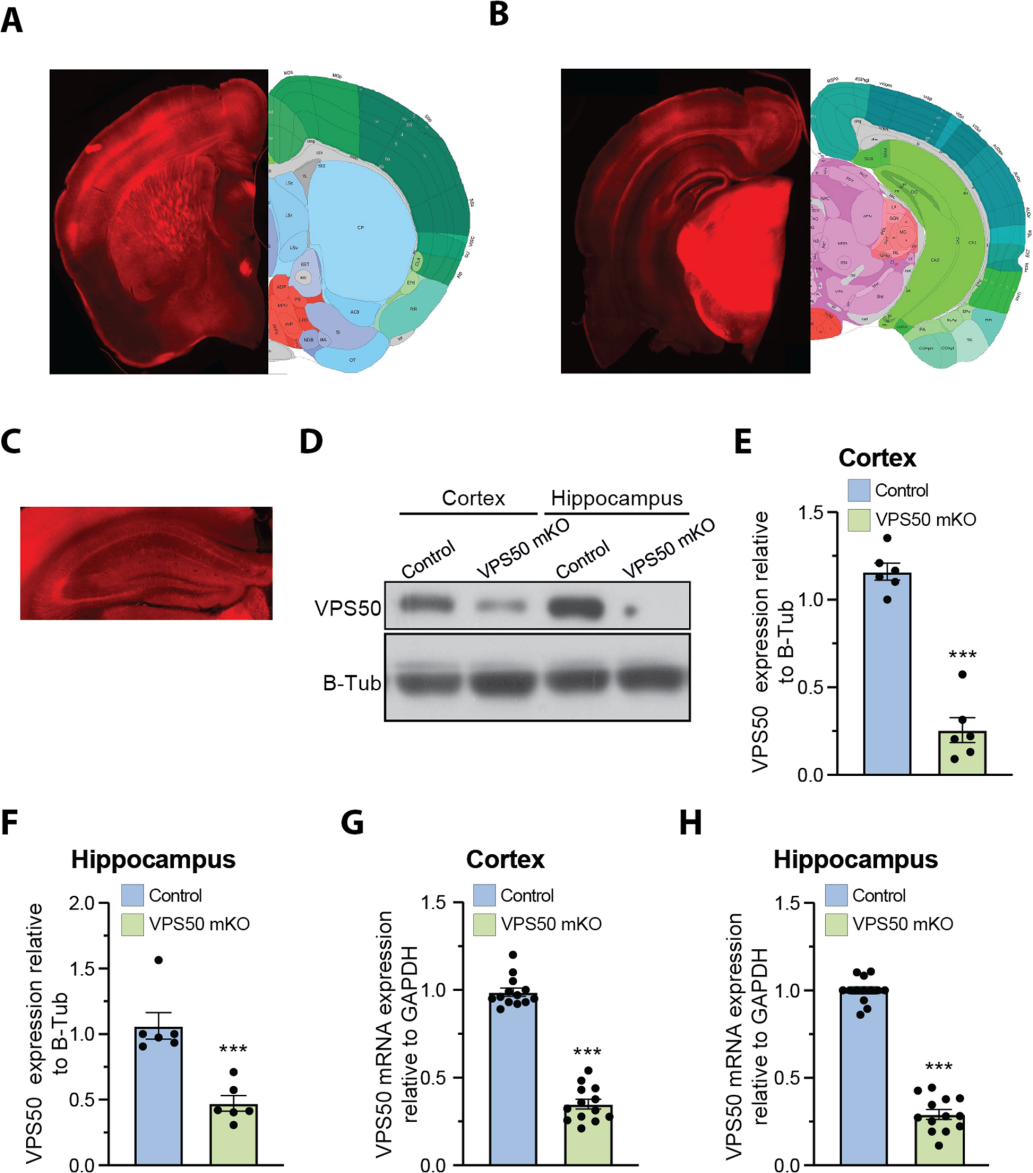
Systemic injection of AAV at P1 to produce VPS50 mKO. **A**, **B** Representative image of coronal sections of a mouse brain (VPS50 mKO) injected at P1 with AAV after 15 weeks to show infection efficiency by tdTomato fluorescent signal. Two different sections are shown to show broad infection. **A**, **B** (right) Anatomical annotations from the Allen Reference Atlas – Mouse Brain (atlas.brain-map.org), at the same slice position as A and B (left). Scheme of the relative positions are shown to identify brain areas. **C** Higher magnification image of a hippocampus section showing transduction efficiency. **D** Representative western blot analyses for the detection of VPS50 expression in control and VPS50 mKO animals in both cortex and hippocampus. B-tubulin was used as loading control. **E**, **F** Quantification of VPS50 expression in western blots from cortex (**E**) or hippocampus (**F**) *n* = 6 animals per condition. **G**, **H** RT-qPCR to quantify expression of VPS50 in cortex (**G**) or hippocampus (**H**), *n* = 13 animals per condition. Scale bar, 500 μm. Unpaired *t*-test was used for statistical analysis; ***p < 0.001. Error bars represent ± SEM

**Fig. 5:**
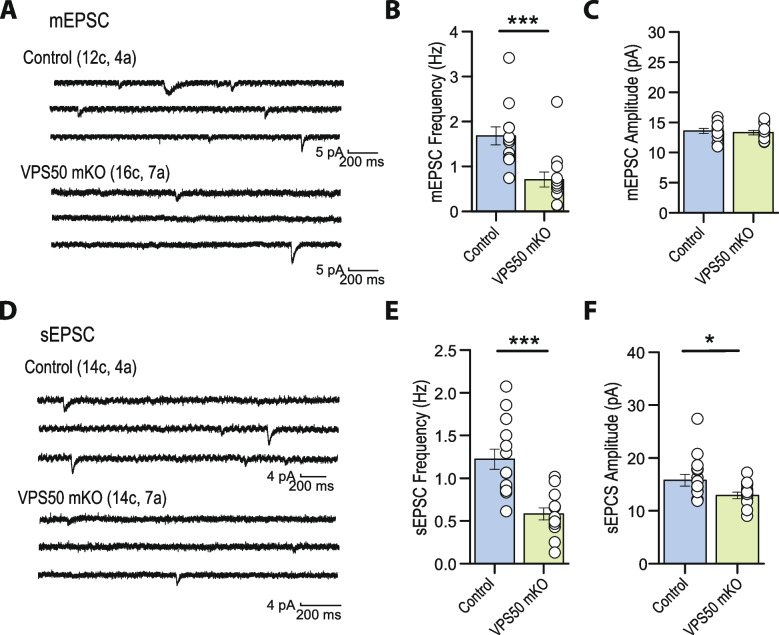
Hippocampal spontaneous excitatory synaptic activity is impaired in VPS50 mKO mouse. **A** Representative traces of mEPSCs recorded from CA1 pyramidal neurons in acute hippocampal slices of control and VPS50 mKO animals. **B**, **C** Quantification of the frequency (**B**) and amplitude (**C**) of mEPSCs. **D** Representative traces of sEPSCs in hippocampal slices of control and VPS50 mKO animals. **E**, **F** Quantification of the frequency (**E**) and amplitude (**F**) of mEPSCs. In each representative trace (**A**–**D**), the numbers of cells (c) and animals (a) are indicated in parentheses. Two-samples Student *t*-test was used for statistical analysis; *p < 0.05, ***p < 0.001. Summary data consist of mean ± SEM

**Fig. 6:**
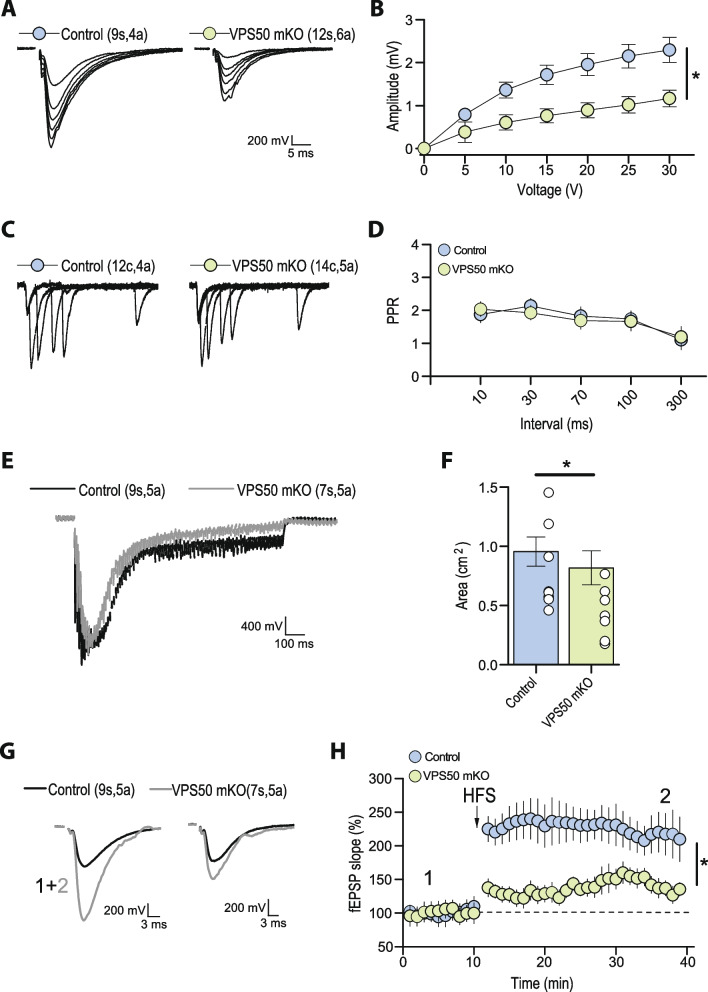
Hippocampal synaptic function and plasticity are impaired in VPS50 mKO mice. Electrophysiological recordings characterizing Schaffer collateral-to-CA1 synapses in acute hippocampal slices of control and VPS50 mKO animals. **A** Representative traces fEPSP of input–output curve elicited at different stimulus intensities. **B** Input–output curves reveal a strong reduction in the amplitude of fEPSC at all stimulus intensities tested. **C** Representative traces and **D** quantification of paired-pulse facilitation at different inter-stimulus intervals. **E** Representative synaptic responses and **F** quantification showing a strong synaptic depression in response to a single high-frequency stimulus train (100 pulses at 100 Hz) that likely reflect a reduce vesicular content in VPS50 mKO synapses compared to control synapses. **G** Representative traces before and after LTP induction elicited by four trains of high-frequency stimulation. Sample traces were taken at times indicated by numbers in summary plot. **H** Summary plot showing that the magnitude of LTP is reduced in VPS50 mKO animals. In each representative trace, the number of slices (s) or cells (c) and animals (a) are indicated in parenthesis. Two-samples Student *t*-test was used for statistical analysis; *p < 0.05. Summary data consist of mean ± SEM

Having demonstrated that VPS50 KO causes a deficit in vesicle acidification as shown by Ratio-SyPhy (Fig. 1G, H), and synaptic function in cultured cortical neurons (Fig. 3), we next aimed to evaluate whether VPS50 mKO in mice can cause changes in synaptic transmission and behavior in vivo, as observed previously for *C. elegans* [14] and as suggested for humans [18]. Toward this end, we used the CRISPR/Cas9 system to induce VPS50 mKO in the intact mouse brain. We delivered high titer AAVs at postnatal day 1 (P1) intracerebroventricularly to Cas9 KI mice. Animals were injected with the same sgRNAs tested previously in culture together with tdTomato as a fluorescence marker under the control of the human synapsin1 promoter; tdTomato alone was used as control. Post-mortem analyses showed high AAV infection in multiples brain areas (Fig. 4A, B), including the cortex and the hippocampus (Fig. 4C). In those brain areas, VPS50 protein expression was significantly reduced > 70% (Fig. 4D–F), confirming efficient mKO of VPS50 after gene editing in the mouse brain. In addition, the expression of VPS50 mRNA was also significantly reduced in VPS50 mKO animals (Fig. 4G, H). No changes were observed in the brain length of VPS50 mKO mice; however, a significant difference in brain weight was found compared to the control group (Additional file 1: Fig. S4A-D). We then monitored basal synaptic function at Schaffer collateral-to-CA1 synapses in acute hippocampal slices. We observed a significant decrease in the frequency but not in the amplitude of miniature excitatory postsynaptic currents (mEPSCs) in VPS50 mKO synapses (Fig. 5A–C). Similarly, we observed a strong reduction in the frequency and amplitude of spontaneous EPSCs (sEPSCs) in VPS50 mKO synapses (Fig. 5D–F). Moreover, input/output curves revealed a large decrease in the amplitudes of evoked EPSC at all stimulus intensities tested for VPS50 mKO synapses (Fig. 6A, B). Importantly, paired-pulse facilitation remained unchanged (Fig. 6C, D), suggesting that the decrease in evoked EPSC amplitude cannot be explained by changes in release probability, but rather could be a consequence of a decrease in vesicle content and/or vesicle refilling caused by a change in the level of acidification, as observed for cultured neurons (Fig. 1G). To further evaluate whether the filling state of vesicles might be involved in this synaptic change, we used high-frequency stimulation to assess the response of VPS50 mKO synapses. High-frequency stimulation promotes full fusion of synaptic vesicles and posterior fast endocytosis for neurotransmitter filling. Thus, during high-frequency stimulation, a neuron with partial or null synaptic vesicle filling would be detected as a decrease in quantal size. Under this condition, the responses of VPS50 mKO to high-frequency stimulation synapses were greatly reduced compared to control synapses (Fig. 6E, F), strongly suggesting that vesicle content is reduced in VPS50 mKO synapses. Next, we investigated the impact of VPS50 mKO on hippocampal long-term synaptic plasticity (LTP), the cellular mechanism underlying learning and memory. We found that the magnitude of LTP induced by high-frequency stimulation was reduced in mice with disrupted VPS50 compared to controls (Fig. 6G, H), suggesting that there might be cognitive deficits in the context of memory formation in VPS50 mKO mice.

**Fig. 7:**
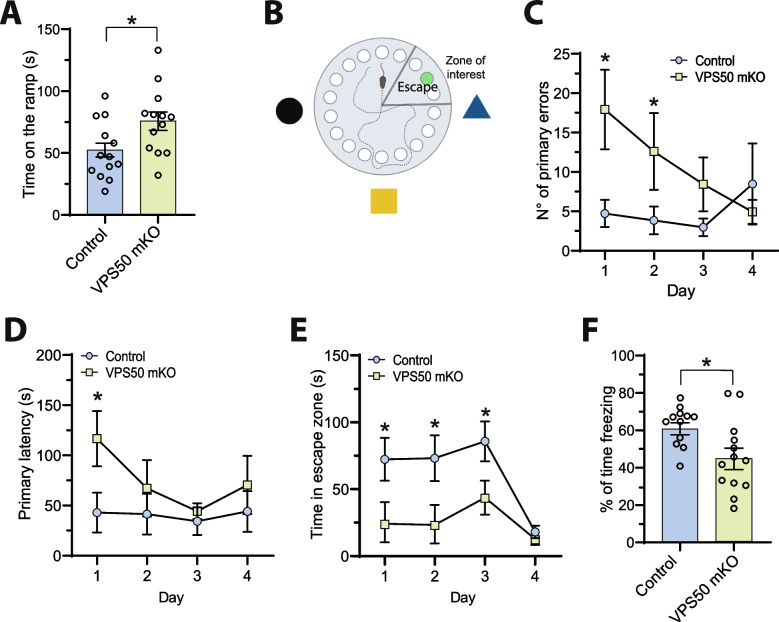
VPS50 mKO animals show impaired hippocampal memory formation. Brain-wide control and VPS50 mKO animals were subjected to behavioral testing. **A** Quantification of the time spent on the ramp on the accelerated rotarod apparatus. **B** Scheme of the Barnes maze memory paradigm in which clues attached to the wall, escape hole, and escape zone are shown. **C** Quantification of the numbers of primary errors made by control and VPS50 mKO animals before finding the escape hole. **D** Quantification of the primary latency of control and VPS50 mKO animals to find the escape hole. **E** Quantification of the time control and VPS50 mKO animals spent in the escape zone. **F** Quantification of freezing percentage time shown by control and VPS50 mKO animals in the fear conditioning paradigm. For behavioral experiments, a total of *N* = 13 animals per condition were assayed. Unpaired *t*-test was used for statistical analysis; *p < 0.05. Error bars represent ± SEM

Finally, we evaluated the cognitive performance of VPS50 mKO mice in the Barnes maze and a fear-conditioning apparatus (Fig. 7), two memory formation paradigms dependent on the hippocampus. First, we asked if VPS50 mKO mice had locomotor problems that might affect their performance in behavioral testing. Accelerated rotarod test showed that VPS50 mKO animals spent more time on the ramp, evidencing better motor function than control animals (Fig. 7A). In the Barnes maze (Fig. 7B), VPS50 mKO animals made a significantly higher number of primary errors (holes checked before finding the escape hole) on days 1 and 2 (Fig. 7C) but behaved similarly to control error numbers on days 3 and 4. Also, primary latency, the time the animal takes to find the escape hole, was significantly affected in VPS50 mKO, but only on the first day (Fig. 7D), and VPS50 mKO animals spent significantly less time in the escape zone on days 1–3 compared to control littermates (Fig. 7E). These data indicate that VPS50 mKO animals have deficits in the short-term acquisition of spatial learning. Using the context-dependent fear-conditioning paradigm, we found that VPS50 mKO animals significantly decreased in freezing compared to control littermates (Fig. 7F). Together, these results indicate that VPS50 mKO mice display alterations in hippocampal synaptic function and memory deficits dependent on this brain area.

## Discussion

The VPS50 protein is evolutionarily conserved, with homologs in *C. elegans, M. musculus*, and humans, reflecting its fundamental importance in cellular function [14, 18]. VPS50 has been associated with the EARP complex (endosome-associated recycling protein) and more specifically with dense core and synaptic vesicles [20]. Studies of *C. elegan*s revealed that VPS50 controls locomotion and behavioral state by controlling vesicle acidification and function, likely by regulating the assembly of the V-ATPase pump [14]. The V-ATPase pump is recruited to synaptic vesicles after recycling to facilitate neurotransmitter filling [21]. *C. elegans* mutants in the gene unc-32, which encodes the homolog of the human V-ATPase pump, have severe deficits in motoneuron neurotransmission [22]. Interestingly, the V-ATPase pump acidifies cellular compartments broadly in the cell; specificity is conferred by the differential formation of the complex by different isoforms and protein interactions [23].

Our findings reveal a critical role of VPS50 in synaptic function and behavior in mammals and demonstrate that this role likely involves synaptic vesicle acidification. Using CRISPR/Cas9 to produce KO of VPS50 in cultured cortical neurons, we showed that VPS50 KO neurons exhibited mislocalization of the V-ATPaseV1 domain, lacking proximity to synaptic vesicles, without affecting the total number of synaptic vesicles. Instead, this mislocalization impedes the correct assembly of the v-ATPase complex affecting vesicle acidification, thereby influencing vesicular content and/or vesicle refilling. The limited acidification of synaptic vesicles is known to reduce neurotransmitter filling and cause a consequent decline in synaptic activity [10, 21, 24]. Our findings strongly indicate that VPS50 plays a crucial role in facilitating the proper sorting of the v-ATPaseV1 domain near synaptic vesicles or the assembly of the v-ATPase complex that permits synaptic vesicle acidification.

Our data further demonstrate that in cultured neurons the reduction of VPS50 impairs synaptic transmission and that these deficiencies can be recovered by artificially acidifying synaptic vesicles. Electrophysiological recordings using hippocampal slices showed a strong decrease in the frequency of spontaneous synaptic events, a reduction of the amplitude after high-frequency stimulation, and a severe deficit in LTP formation. These observations strongly support the idea that the changes observed in synaptic activity were a consequence of the exocytosis of empty or partially filled synaptic vesicles, similar to what has been shown in cultured hippocampal neurons in which the v-ATPase pump had been pharmacologically blocked [25]. These synaptic transmission defects likely caused the impaired memory formation of VPS50 mKO animals, linking the deficits in synaptic vesicle acidification and/or synaptic filling to complex cognitive behaviors such as learning and memory formation. Deficits in working and spatial memory have been described as one of the phenotypes of ASD patients [8, 9], a phenotype that has also been described for patients with VPS50 mutations [18].

In summary, our data demonstrate that VPS50 modulates synaptic transmission by facilitating the recruitment of the V-ATPase pump to synaptic vesicles, thereby enabling vesicle acidification and modulating vesicle content. Deficits in this process, as exhibited by VPS50 mKO mice, result in cognitive impairment. Future studies of VPS50 mKO mice should provide a comprehensive understanding of the behavioral consequences of VPS50 mKO and provide insights into how mutations in the VPS50 gene can lead to ASD.

## Conclusions

Our study provides compelling evidence for the critical role of the evolutionarily conserved VPS50 protein in synaptic function and behavior across mammalian species. Utilizing CRISPR/Cas9 knockout techniques, we have elucidated the involvement of VPS50 in regulating synaptic vesicle acidification, a process crucial for neurotransmitter filling and synaptic activity. Our findings shed light on the molecular mechanisms underlying VPS50’s function and highlight the significance of VPS50 in maintaining synaptic transmission integrity, as evidenced by the impaired synaptic activity and cognitive deficits observed in VPS50 mKO mice. These results not only deepen our understanding of synaptic function but also provide valuable insights into the etiology of neurodevelopmental disorders such as ASD.

## Methods

### Animals and injections

All animal procedures and experiments were performed according to NIH and ARRIVE guidelines and were approved by MIT Committee on Animal Care (CAC) and the animal ethics committee from Universidad Andrés Bello (020/2018). Animal injections were performed as in [26, 27]. Briefly, newborn Cas9 KI mice (C57BL/6J; JAX 026179) were cryoanesthetized in a cold aluminum plate and injected with 1 μL of concentrated AAV (1 × 10^11^ vg) in each cerebral ventricle at a depth of 3 mm in the animal’s head at 2/5 of the intersection between lambda and the eye with a 10-μL HAMILTON syringe (Hamilton, 7653–01) and a 32-G needle (Hamilton, 7803–04). After the injection, mice were placed in an infrared heating pad at 37 °C until they recovered their pink color, at which point they were returned to the cage with their mother [26–28]. In the third week after birth, mice were weaned and separated by sex into cages with a 12 h/12 h light/dark cycle with free access to food and water. An electronic chip (p-chips, Pharmaseq) with a unique identifier was put in the tail of each injected animal to facilitate animal tracking. After behavioral testing, mice were anesthetized with isoflurane (3% in 0.8 L/min air flow) in an induction chamber for 3 min and decapitated for molecular analyses.

### Neuronal cultures

At P1, neonatal mice were quickly decapitated and dissected in ice-cold HBSS to obtain cerebral cortices as described by [26, 27, 29, 30]. Cortices were minced and incubated for 20 min at 37 °C with papain (Worthington, USA) for enzymatic digestion. Then, cells were transferred to a 15-ml tube containing plating media (D-MEM supplemented with 10% fetal bovine serum and 100 U/ml penicillin/streptomycin; Life technologies 15,070–063). Cells were resuspended by mechanical agitation through fire-polished glass Pasteur pipettes of decreasing diameters. Cells were plated on freshly prepared poly-L-lysine-coated plates. After 2 h, plating media were replaced with growth media (Neurobasal-A (Life technologies 1,088,802) supplemented with B27 (Life technologies 17,504,044), 2 mM L-glutamine (Life technologies 25,030–081), 100 U/ml penicillin/streptomycin (Life technologies 15,070–063)). Half of the media was replaced every 3 days.

### AAV production

AAV viral particles containing sgRNA directed to VPS50, red fluorescent protein tdTomato under the control of the human Synapsin1 promoter (hSyn), and packaged with the PHP.eB capsid [31] were obtained from HEK 293T cells and purified as described by [26, 27]. AAVs were also prepared using transfer plasmids encoding GCamP7f to measure calcium transients (Addgene Cat#104,488), and custom AAV plasmids for enhanced Cas9 (eCas9) [32], SyPhy to assess synaptic vesicle acidification [33], plus pHoenix to artificially acidify synaptic vesicles [19]. Briefly, HEK 293T cells were grown to approximately 6 × 10^4^ cells/cm^2^ with DMEM 10% FBS. Cultures were transfected using the PEI “MAX” reagent (Polysciences, Cat 24,765) with PHP.eB capsid plasmids, the vector with transfer plasmids, and the helper plasmid DF6. After 24 h of transfection, the media was exchanged for DMEM 1% FBS. After 72 h, media was collected from the plates and replaced with fresh DMEM 1% FBS. The collected media was stored at 4 °C, and 120 h after transfection cells were detached from the plate and transferred to 250-mL conical tubes, together with the collected media and centrifuged for 10 min at 2000*g*. The supernatant was removed and saved for later use, and the pellet was resuspended in SAN digestion buffer (5 mL of 40 mM Tris, 500 mM NaCl, and 2 mM MgCl2 pH 8.0) containing 100 U/mL of Salt Active Nuclease (Arcticzymes, USA) and incubated at 37 °C for 1 h. The supernatant was precipitated using 8% PEG 8000 and 500 mM NaCl, incubated on ice for 2 h, and centrifuged at 4000*g* for 30 min in 250-mL bottles. Pellet was resuspended with SAN digestion buffer and the complete solution was placed in an iodixanol gradient (15 to 60%) and centrifuged at 350,000*g* for 2.5 h. The phase containing the AAV was rescued and frozen at − 80 °C for later use.

### Proximity ligation assays

Assays were conducted using the Duolink PLA Fluorescence kit (Sigma-Aldrich) following the manufacturer’s instructions. Briefly, cortical cultured neurons were fixed using 4% paraformaldehyde for 20 min at RT, washed 3 times with PBS, permeabilized using 0.05% Triton X-100 for 5 min, and rinsed 3 times with PBS. Samples were incubated with blocking solution for 1 h at 37 °C and incubated with primary antibodies (VPS50, Sigma Aldrich, HPA026679, rabbit 1:100; PSD95, Neuromabs, 75–348, mouse 1:250; Synapsin1, Synaptic Systems, 106,011, mouse 1:1000; Synaptophysin, Zymed, 180,130, rabbit 1:1200; v-ATPase H, Santa Cruz Biotechnology, SC-166227, mouse 1:100) overnight at 4 °C. Samples were washed twice with buffer A for 5 min, incubated with plus-minus probes for 1 h at 37 °C, washed twice with buffer A, and incubated with ligation mixture for 30 min at 37 °C. Samples were washed twice with buffer A, incubated with an amplification mixture for 2 h at 37 °C, and washed twice for 10 min with buffer B. Lastly, samples were mounted, and images were acquired using a Nikon C2 + confocal microscope (Nikon, USA).

### Brain sectioning and mounting

To assess brain infection, half of the brains were submerged in PBS + 4% PFA + 4% Sucrose into 30-mL flasks and fixed for a minimum of 24 h. After fixation, a Leica VT1000 vibratome was used to cut 100-μm coronal sections. Slices were kept in PBS 1X and mounted using Fluoromount G (EMS, Hatfield, PA) to preserve the fluorescence signal. Brain images were captured with a Nikon Eclipse TE2000 epifluorescence microscope (Nikon, USA).

### Protein extraction and electrophoresis

For immunodetection tests, proteins were extracted from the cortex and hippocampus of mice brains. Fifty milligrams of tissue was ground in N-PER lysis buffer (ThermoFisher Product No. 23225) with protease and phosphatase inhibitors (cOmplete Protease Inhibitor Cocktail 11,697,498,001, Roche Diagnostics) until a homogeneous solution was achieved and incubated for 10 min on ice. This extract was centrifuged at 10,000*g* for 10 min at 4 °C. The supernatant with extracted proteins was collected and quantified using the BCA method, following the manufacturer’s recommendations (Perkin-Elmer). For electrophoresis, the proteins were denatured in loading buffer (NuPAGE LDS Sample buffer 4X; NP0007) and heated at 95 °C for 10 min. Forty micrograms of the sample was loaded onto an SDS 6% acrylamide-bisacrylamide gel to visualize proteins larger than 100 kDa and a 10% gel for proteins smaller than 100 kDa. Electrophoresis was performed at 80–150 V constant voltage in running buffer (25 mM Tris·Cl, 250 mM glycine, 0.1% SDS) in a minichamber (MiniPROTEAN System, BIO-RAD).

### Western blots

After electrophoresis, proteins were transferred to an activated PDVF membrane in transfer buffer (250 mM glycine, 25 mM Tris–Cl, 20% methanol) at 400 mA constant current for 1.5 h. To verify protein transfer, the membrane was incubated with Ponceau red S (0.1% Ponceau S, 5% acetic acid). The membrane was washed with TBS/Tween 20 0.05% until the Ponceau red staining was removed. Next, the membrane was blocked for 1 h to eliminate non-specific protein binding sites in a 0.05% TBS/Tween 20 solution with 5% skim milk. The membrane was then incubated with the specific primary antibody VPS50 (Sigma, HPA026679-100UL, Rabbit, 1/500 dilution) in 0.05% TBS/Tween 20 solution with 5% skim milk at 4 °C overnight. The membrane was washed with TBS/Tween 20 0.05% five times for 5 min each. Then, the membrane was incubated with a second antibody directed against the first antibody and coupled to horseradish peroxidase (HRP) (anti-rabbit HRP, CS7074, Cell Signaling Technology, USA) in TBS/Tween 20 0.05% with 5% skim milk at room temperature for 1 h. The membrane was washed with TBS/Tween 20 0.05% five times for 5 min each. Detection was performed with chemiluminescence reagents (SignalFire™ Elite ECL Reagent). Protein expression was normalized to β-Tubulin expression (Abcam, ab6046, Rabbit, 1/500) or N-Cadherin (Santa-Cruz Biotechnology, SC-7939, rabbit. 1/500).

### DNA and RNA extraction

Thirty to 50 mg of tissue were weighed for DNA and RNA extractions with Quick-DNA/RNA Miniprep kit (Zymo Research, D7001). Briefly, the tissue was homogenized with a Dounce homogenizer in lysis buffer and centrifuged for 30 s at 14,000*g*. The supernatant was collected and transferred to a Zymo-Spin IIICR column in a collection tube and centrifuged at 14,000*g* for 30 s. The filtrate was used for RNA extraction, and the column for DNA extraction. For RNA extraction, the same volume of 100% ethanol was added and homogenized, transferred to a Zymo-Spin IICR column, and centrifuged at 14,000*g* for 30 s. The column was then treated with DNase I, 400 μL DNA/RNA Prep Buffer was added, and the column was centrifuged and washed twice with wash buffer. To elute the RNA, 25 μL of nuclease-free water was added, incubated for 3 min, quantified, and stored at − 80 °C for later use. For DNA extraction, 400 μL DNA/RNA Prep Buffer was added to the Zymo-Spin IIICR column, and the column was centrifuged and washed twice with wash buffer. To elute the DNA, 50 μL of nuclease-free water was added, incubated for 5 min, quantified, and stored at − 80 °C for later use.

### RT-qPCR

RT-qPCR assays were performed using 400 ng of extracted total RNA. To obtain complementary DNA (cDNA), each sample was mixed with 0.25 μg Oligo-dT (New England Biolab S1316S) in 10 μL of nuclease-free water, denatured at 75 °C for 5 min and then quickly transferred to 4 °C for 5 min. Reverse transcription (RT) was performed in a final 20 μL volume containing: 10 μL denatured RNA, 100 U of M-MLV Reverse Transcriptase (NEB; M0253S), M-MLV RT buffer (NEB; B0253S) 1X, 20 U of RNase Inhibitor (NEB; M0314S), and 0.5 mM of dNTPs (Biotechnology N557-0.5ML). The mixture was incubated at 42 °C for 1 h, then at 95 °C for 5 min, and the reaction was stopped at 4 °C and then diluted five times with nuclease-free water. Three microliters of the diluted cDNA were used for real-time PCR reactions, using relative abundance by the ddCt method, and GAPDH transcript as a loading control. Transcript detection was performed with specific primers for VPS50 mRNA (Sense: TGTTACTTCTCCGAGGCAGG, Antisense: GCTCTCAAAGGACCAAGAT) and GAPDH mRNA (Sense: ATGGTGAAGGTCGGTGTGAA, Antisense: CATTCTCGGCCTTGACTGTG).

### Transmission electron microscopy

Electron microscopy was conducted as described in [34]. Briefly, cortical neurons were grown on sterilized Aclar pieces (EMS, Cat#50,425) and infected at 3 DIV with AAVs to produce VPS50 KO. Ten days after infection, cultures were fixed with 4% Glutaraldehyde in 0.1M Cacodylate Buffer for 1 h at RT, and a secondary fix in 1% Osmium tetroxide in 0.1M Cacodylate buffer on ice 1 h. Samples were dehydrated using increasing concentrations of ethanol and embedded in Epon resin using an aluminum weighing dish at 60 °C for 48 h. After polymerization the resin was taken out of the aluminum dish, pieces were sawed out and mounted for sectioning at 50 nm and imaged using a transmission electron microscope (JEOL JEM-1200 ExII) and CCD camera (AMT XR-41).

### Calcium imaging

Control and VPS50 KO cortical neurons were co-infected at 3 DIV with GCaMP7f. At 10 days in vitro (DIV), neurons were imaged using a Nikon Eclipse TE-2000 microscope (Nikon, USA) equipped with a CO2/temperature chamber (Tokai-Hit). Pictures were acquired every 30 ms for 5 min. Using ImageJ (NIH) regions of interest were selected in neuronal somas to determine fluctuations of fluorescence over time to calculate calcium transient frequency. The Kolmogorov–Smirnov test was used to test for significance.

### Electrophysiology of cultured neurons

Whole-cell patch-clamp recordings of infected cortical neurons were performed and analyzed as previously described [27, 30, 35]. The external solution contained 150 mM NaCl, 5.4 mM KCl, 2.0 mM CaCl2, 2.0 mM MgCl2, 10 mM HEPES (pH 7.4), and 10 mM glucose. Patch electrodes (5–7 MΩ) were filled with 120 mM CsCl, 10 mM BAPTA, 10 mM HEPES (pH 7.4), 4 mM MgCl2, and 2 mM ATP-Na2. After the formation of a high resistance seal and break-in (> 1 GΩ), whole-cell voltage and current signals were recorded with an Axopatch 700B amplifier (Molecular Devices). Signals were low-pass filtered (5 kHz) and digitized (5–40 kHz) using a PC with pClamp10 software. Cells were held at − 60 mV.

To analyze synaptic function, we recorded isolated AMPA-mediated synaptic currents using a mixture of antagonists against NMDA receptors (NMDARs; 20 μM d-APV) and GABA_A_ receptors (GABAARs; 2 μM bicuculline). In some experiments, the sodium channel blocker tetrodotoxin (TTX; 500 nM) was added to the bath to record miniature AMPA-mediated currents. For current-clamp analyses, cells were patched, and spontaneous spiking of cells was recorded. MiniAnalysis software (Synaptosoft) was used to analyze synaptic events. The frequency and amplitude of currents were automatically calculated and plotted.

### Hippocampal slice electrophysiology

Electrophysiological recording from hippocampal slices was conducted as previously described [27, 30, 36–38]. Briefly, acute coronal hippocampal slices (400 mm thick) were prepared from control and VPS50 mKO mice at postnatal day (P) 30 to P45. Mice were anesthetized with isoflurane (3% in 0.8 L/min air flow) in an induction chamber for 3 min and decapitated. Brain slices were cut using a DKT vibratome in a solution containing the following: 215 mM sucrose, 2.5 mM KCl, 26 mM NaHCO3, 1.6 mM NaH2PO4, 1mM CaCl2, 4 mM MgCl2, 4 mM MgSO4, and 20 mM glucose. After 30-min recovery, slices were incubated in an artificial CSF (ACSF) recording solution containing the following: 124 mM NaCl, 2.5 mM KCl, 26 mM NaHCO3, 1 mM NaH2PO4, 2.5 mM CaCl2, 1.3 mM MgSO4, and mM 10 glucose equilibrated with 95% O2/5% CO2, pH 7.4. Slices were incubated in this solution for 30 min before recordings.

Except as otherwise indicated, all electrophysiological experiments were performed at 28 ± 1 °C using a submersion-type recording chamber perfused at 1–2 ml/min rate with ACSF supplemented with the GABA_A_ receptor antagonist picrotoxin (PTX; 100 μM). Extracellular field potentials (fEPSPs) were recorded with a patch pipette filled with 1 mM NaCl and placed in the CA1 stratum radiatum. Whole-cell voltage-clamp recordings (Multiclamp 700B Molecular Devices, USA) were made from CA1 pyramidal neurons voltage-clamped at − 60 mV using patch pipette electrodes (3–4 MΩ) containing the following intracellular solution: 131 mM Cs-gluconate, 8 mM NaCl, 1 mM CaCl2, 10 mM EGTA, 10 mM glucose, 10 mM HEPES, pH 7.2, 292 mmol/kg osmolality. fEPSPs and EPSCs were evoked by stimulating Schaffer collateral inputs with a monopolar electrode filled with ACSF and positioned ~ 100–150 μm away from the recording pipette. mEPSCs were recorded at 32 ± 1 °C in the continuous presence of tetrodotoxin (TTX, 500 nM) to block action potential-dependent release, whereas sEPSCs were recorded in the absence of TTX. Short-term synaptic plasticity was induced by two pulses (100 ms interstimulus interval) to calculate a paired-pulse ratio (PPR) that was defined as the ratio of the slope or amplitude of the second fEPSP/EPSC to the slope or amplitude of the first fEPSP/EPSC, respectively. Long-term potentiation (LTP) was induced by 4 trains of 100 pulses at 100 Hz repeated four times, separated by 10 s. The magnitude of LTP was compared with baseline 30–40 min after applying the stimulation protocol. Reagents were obtained from Sigma, Tocris, and Ascent Scientific; prepared in stock solutions (water or DMSO); and added to the ACSF as needed. Total DMSO in the ACSF was maintained at less than 0.01%. For the whole-cells experiments, series resistance (range, 8–12 MΩ) was monitored throughout the experiment with a 5 mV, 80 ms voltage step, and cells that exhibited significant changes in series resistance (20%) were excluded from analysis. All recordings were elicited at 15–20-s intervals, filtered at 2.4 kHz, and acquired at 10 kHz, using custom-made software written in Igor Pro 6.36 (WaveMetrics). Statistical comparisons were made using two samples Student’s *t*-test at *p* < 0.05 significance level, using Origin 9.3 Pro software (OriginLab). Unless otherwise indicated, all slice electrophysiological values are provided as mean ± S.E.M and illustrated traces are average of 15–20 responses.

### Behavioral analyses

All behavioral tests of mice were performed 8 weeks after AAV injection and conducted as previously described [26, 27, 30, 38]. Before each test, mouse cages were transported to the behavior room and habituated for 30 min in the dark. After completing a trial, the equipment and devices used were cleaned with 70% ethanol. Tests were recorded and analyzed with ANY-Maze software (Stoelting, USA). Behavior tests were performed between 9:00 am and 6:00 pm. At the end of the battery of behavioral tests, the animals were sacrificed for subsequent molecular analyses.

#### Contextual fear conditioning

An UGO-BASILE apparatus controlled by ANY-Maze was used. This equipment consists of a sound attenuating box, fan, light (visible/IR), a speaker, a USB camera, a single onboard controller, and a mouse cage. All trials were recorded, and all mice underwent habituation, conditioning, and testing phase. Twenty-four hours after training, the animals were tested for contextual memory. Each mouse was placed in the fear-conditioning box, allowed to explore for 5 min freely, and returned to its cage. The number of freezing episodes and freezing time were recorded.

#### Barnes maze

A non-reflective gray circular platform (91 cm diameter) with 20 holes (5 cm diameter) evenly distributed along the perimeter, with one hole containing a metal escape tunnel, was used. Three exogenous visual cues (length/width ~ 30 cm) were used around the platform: a black circle, a blue triangle, and a yellow square. The light was adjusted to 1000 lx in the center of the platform. All animals underwent a phase of habituation, spatial acquisition, and testing. On test day, the position of the escape tunnel was changed, and the animal was brought in the start box to the center of the platform, left for 10 s, and sound reproduction was started. The test ended at 90 s or when the mouse found the escape tunnel. The number of primary and total errors, primary and total latency, and total distance before finding the gap were recorded. The number of visits to each hole was also measured to show preference.

### Data analysis

All values are presented as means ± standard errors (SE) for three or more independent experiments. Most statistical analyses were performed using the Student’s *t*-test, whereas slice electrophysiological data were compared using two-tailed Student’s *t*-test. For calcium imaging analyses, the Kolmogorov–Smirnov test was used to test for significance. Values of *p* < 0.05 are considered statistically significant. Statistical analyses were performed using OriginPro 9.3 software (OriginLab Corporation) or Graphpad Prism 10 (Dotmatics).

### Supplementary Information

**Additional file 1: Fig. S1.** Cortical neurons were infected at 3 DIV with AVV encoding TdTomato as control and a combination of sgRNAs (1–6 / 2–4 / 1–4 / 2–6) targeting Vps50, as indicated. 10 days later genomic DNA was extracted, and the T7 endonuclease I assay was performed. (A) T7 endonuclease I assay for sgRNA combinations showing successful editing of the VPS50 locus with all sgRNA combinations as shown by lower size bands in the treated samples. (B) Sequences of VPS50 locus edited with sgRNAs in VPS50 KO cultured neurons showing the changes in the DNA sequence after gene editing using locus-specific sequencing. **Fig S2.** Proximity ligation assay (PLA) in Control and VPS50 KO neurons. PLA was performed using specific antibodies to determine proximity between proteins of interest. Low magnification (20x) for each condition are shown. Microtube-associated protein 2 (MAP2) was used to stain all neurons. (A) PLA for Synapsin1 (Syn1)-Synaptophysin (Syn) (pre-presynaptic) or (B) Synaptophysin-PSD95 (pre-post synaptic). Reaction controls where PLA signal was observed only for the Synapsin1-Synaptosophysin pair, the two proteins known to be in proximity. Lower magnification of PLA reactions in Fig. 2 to show different combinations of PLA reactions to determine proximity between (C) VPS50/PSD95, (D) VPS50/Syn1, (E) VPS50/v-ATPaseV1, (F) VPS50/v-ATPaseV1 in VPS50 KO neurons, (G) v-ATPaseV1/Syn, and (H) v-ATPaseV1/Syn in VPS50 KO neurons. (I) PSD95 and Synaptophysin protein expression in VPS50 KO and control neurons. N-cadherin was used as loading control. Scale bars, 25 μm. **Fig. S3.** VPS50 KO neurons show deficits in synaptic transmission. (A) Representative traces of AMPA-mediated EPSCs followed for 30 min for control and VPS50 KO neurons. VPS50 KO neurons show a robust reduction in both Amplitude (B) and Frequency (C) of AMPA EPSCs. (D-E) Cortical neurons were co-transduced with GCaMP7 to measure calcium events by changes in fluorescence over time. (D) Representative traces and quantification (E) of changes of fluorescence over time in control and VPS50 KO neurons. A significant reduction is observed in VPS50 KO neurons compared to control. Control *n* = 1214; VPS50 mKO *n* = 1175 cells from 3 independent experiments; Kolmogorov–Smirnov test was used to test for significance. For electrophysiology recordings at least 18 neurons from 3 independent experiments were analyzed. Unpaired t-test was used for statistical analysis; *p < 0.05, ***p < 0.001. Error bars represent ± SEM. **Fig. S4.** Anatomical changes of Control and VPS50 mKO brains. (A-B) Representative images of full brains from Control and VPS50 mKO animals. (C-D) Quantification of the total length (C) and weight (D) of brains extracted from Control or VPS50 mKO animals. (E–F) Representative bright field images of brain slices from Control (E) and VPS50 mKO (F) brains.

**Additional file 2. **Original gels/blots.

## Data Availability

All data generated or analyzed during this study are included in this published article and its supplementary information files.

## Abbreviations

ASD: Autism spectrum disorders
CRISPR: Clustered regularly interspaced short palindromic repeats
KO: Knockout
mKO: Mosaic knockout
SNPs: Single nucleotide polymorphism
CNVs: Copy number variants
GARP: Golgi-associated retrograde protein
EARP: Endosome-associated recycling protein
WT: Wild-type
PLA: Proximity ligation assay
DIV: Days in vitro
mEPSC: Miniature excitatory postsynaptic currents
sEPSC: Spontaneous excitatory postsynaptic currents
AAV: Adeno-associated virus
LTP: Long-term potentiation
PPR: Paired-pulse ratio
fEPSPs: Extracellular field potentials
