# Supplementary material for: Deletion of VPS50 protein in mouse brain impairs synaptic function and behavior
Source: BMC Biol. 2024 Jun 26;22:142. doi: 10.1186/s12915-024-01940-y (PMC11210182; doi:10.1186/s12915-024-01940-y)
Supplement: Supplementary file 1 — Additional file 1: Fig. S1. Cortical neurons were infected at 3 DIV with AVV encoding TdTomato as control and a combination of sgRNAs (1–6 / 2–4 / 1–4 / 2–6) targeting Vps50, as indicated. 10 days later genomic DNA was extracted, and the T7 endonuclease I assay was performed. (A) T7 endonuclease I assay for sgRNA combinations showing successful editing of the VPS50 locus with all sgRNA combinations as shown by lower size bands in the treated samples. (B) Sequences of VPS50 locus edited with sgRNAs in VPS50 KO cultured neurons showing the changes in the DNA sequence after gene editing using locus-specific sequencing. Fig S2. Proximity ligation assay (PLA) in Control and VPS50 KO neurons. PLA was performed using specific antibodies to determine proximity between proteins of interest. Low magnification (20x) for each condition are shown. Microtube-associated protein 2 (MAP2) was used to stain all neurons. (A) PLA for Synapsin1 (Syn1)-Synaptophysin (Syn) (pre-presynaptic) or (B) Synaptophysin-PSD95 (pre-post synaptic). Reaction controls where PLA signal was observed only for the Synapsin1-Synaptosophysin pair, the two proteins known to be in proximity. Lower magnification of PLA reactions in Fig. 2 to show different combinations of PLA reactions to determine proximity between (C) VPS50/PSD95, (D) VPS50/Syn1, (E) VPS50/v-ATPaseV1, (F) VPS50/v-ATPaseV1 in VPS50 KO neurons, (G) v-ATPaseV1/Syn, and (H) v-ATPaseV1/Syn in VPS50 KO neurons. (I) PSD95 and Synaptophysin protein expression in VPS50 KO and control neurons. N-cadherin was used as loading control. Scale bars, 25 μm. Fig. S3. VPS50 KO neurons show deficits in synaptic transmission. (A) Representative traces of AMPA-mediated EPSCs followed for 30 min for control and VPS50 KO neurons. VPS50 KO neurons show a robust reduction in both Amplitude (B) and Frequency (C) of AMPA EPSCs. (D-E) Cortical neurons were co-transduced with GCaMP7 to measure calcium events by changes in fluorescence over time. (D) Representative trace [file 12915_2024_1940_MOESM1_ESM.pdf]

A

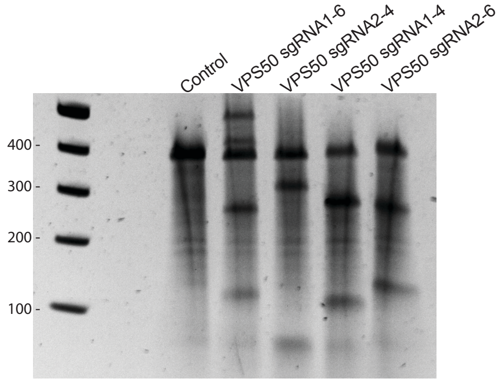

B

Control

GGAAGGCTGACCCAGGCGGGCAGCTCCACGTGACGACTCACTATGGCTTCCCTGTGTTGTAGCCGGCTGTCTGGTGGGATTATGTGATTTGTTACTTCTCCGAGGCAGG

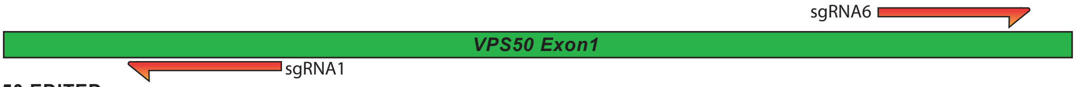

VPS50 EDITED

GGAAGGCTGACCCAGGCG --GGGATTATGTGATTTGTTACTTCTCCGAGGCAGG
GGAAGGCTGACCCAGGCGGGCAGCT --TGTGATTTGTTACTTCTCCGAGGCAGG
GGAAGGCTGACCCAGGCGG --GTGATTTGTTACTTCTCCGAGGCAGG
GGAAGGCTGACCCAGGCGGGC -- ATTATGTGATTTGTTACTTCTCCGAGGCAGG
GGAAGGCTGACCCAGGCGGGCAGCT -- ATTATGTGATTTGTTACTTCTCCGAGGCAGG
GGAAGGCTGACCCAGGC -- TGATTTGTTACTTCTCCGAGGCAGG
GGAAGGCTGACCCAGGCGGGCAGCTCCACGT -- GTGGGATTATGTGATTTGTTACTTCTCCGAGGCAGG
GGAAGGCTGACCCAGGCGGGCAGCTCCAC --GTGGGATTATGTGATTTGTTACTTCTCCGAGGCAGG
GGAAGGCTGACCCAGGCGGGCAGC-- TGTGATTTGTTACTTCTCCGAGGCAGG
GGAAGGCTGACCCAGGCGGGCAGCTCCACG-- GGGATTATGTGATTTGTTACTTCTCCGAGGCAGG
GGAAGGCTGACCCAGGC --TGATTTGTTACTTCTCCGAGGCAGG
GGAAGGCTGACCCAGGCGGGCAGCT -- ATTATGTGATTTGTTACTTCTCCGAGGCAGG
GGAAGGCTGACCCAGGCGGGCAGCT -- ATTATGTGATTTGTTACTTCTCCGAGGCAGG
GGAAGGCTGACCCAGGCGGGCA --TATGTGATTTGTTACTTCTCCGAGGCAGG
GGAAGGCTGACCCAGGCG --GTGATTTGTTACTTCTCCGAGGCAGG
GGAAGGCTGACCCAGGCGGGC --GTGATTTGTTACTTCTCCGAGGCAGG

Ahumada-Marchant et al., Fig. S1.

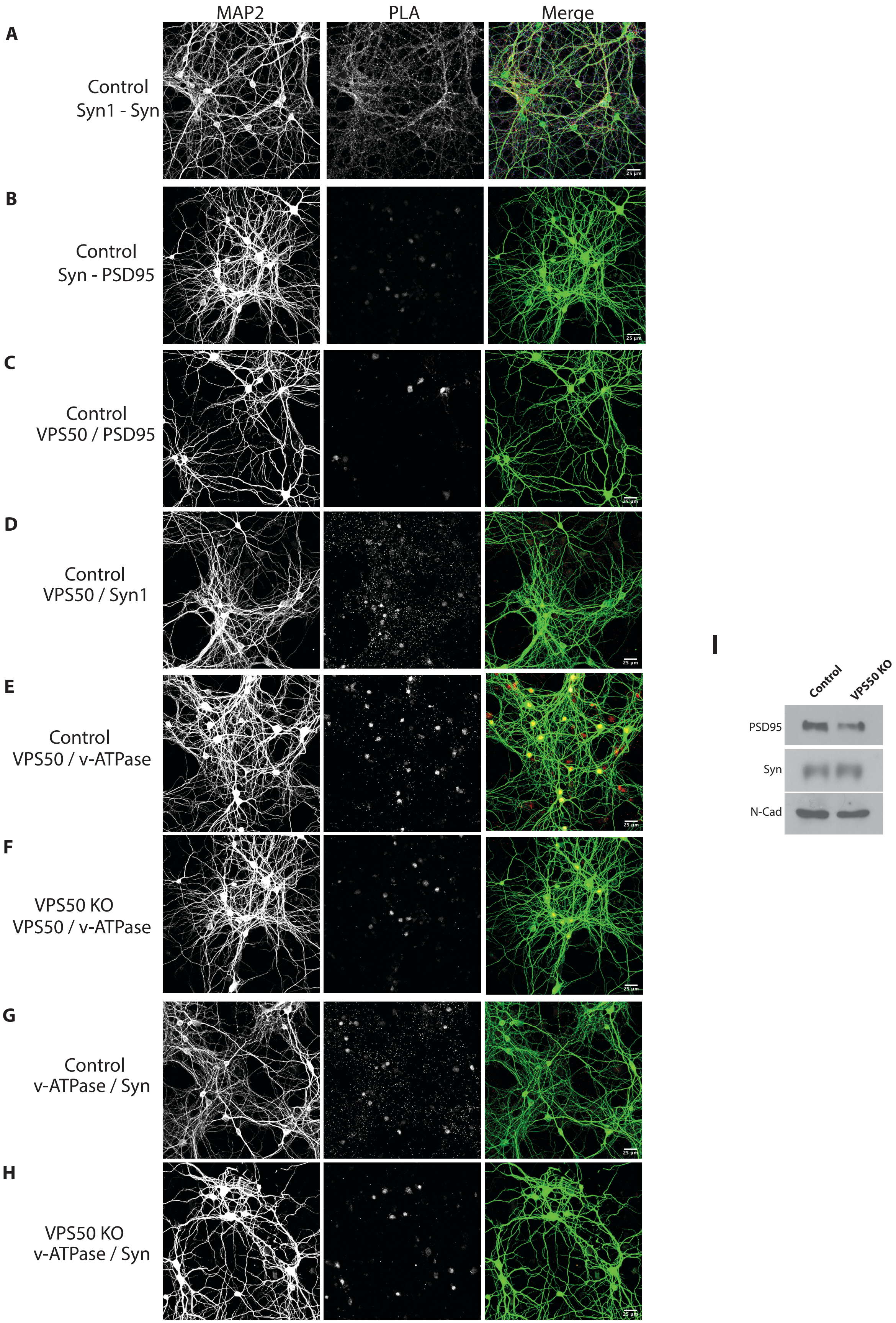

Ahumada-Marchant et al., Fig. S2.

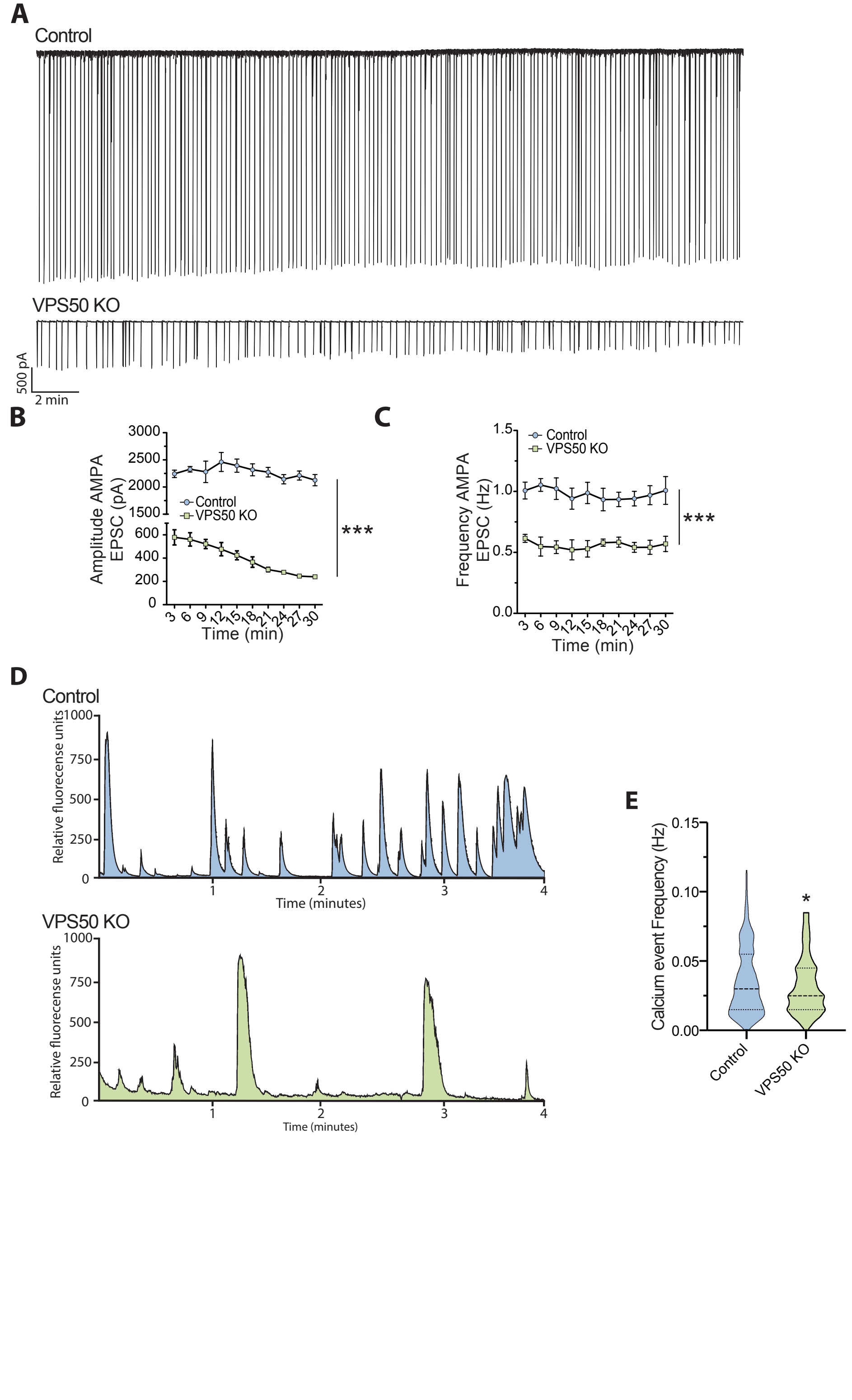

Ahumada-Marchant et al., Fig. S3.

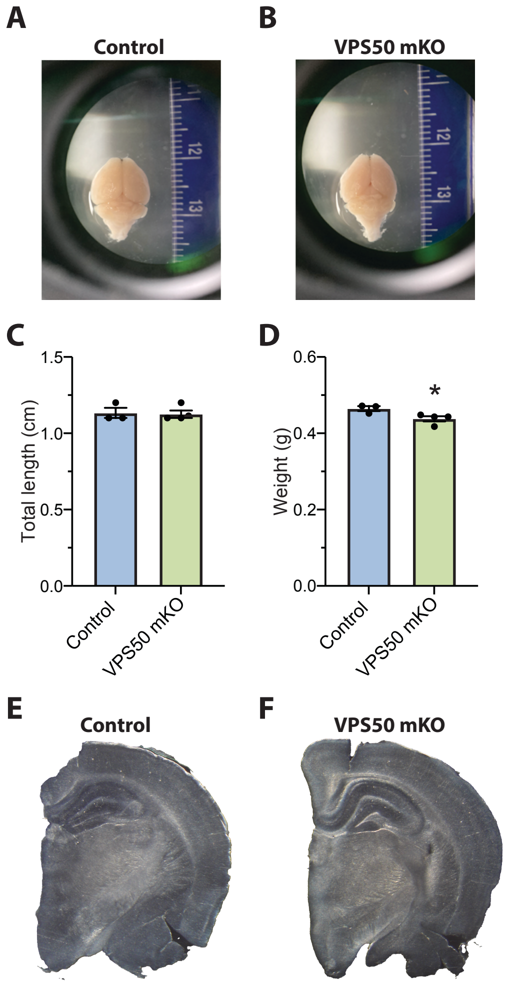

Ahumada-Marchant et al., Fig. S4.
