# Supplementary material for: Deletion of VPS50 protein in mouse brain impairs synaptic function and behavior
Source: BMC Biol. 2024 Jun 26;22:142. doi: 10.1186/s12915-024-01940-y (PMC11210182; doi:10.1186/s12915-024-01940-y)
Supplement: Supplementary file 2 — Additional file 2. Original gels/blots. [file 12915_2024_1940_MOESM2_ESM.pdf]

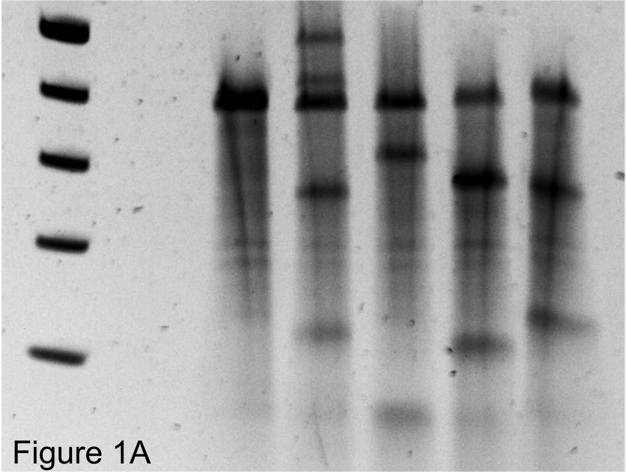

Figure 1A

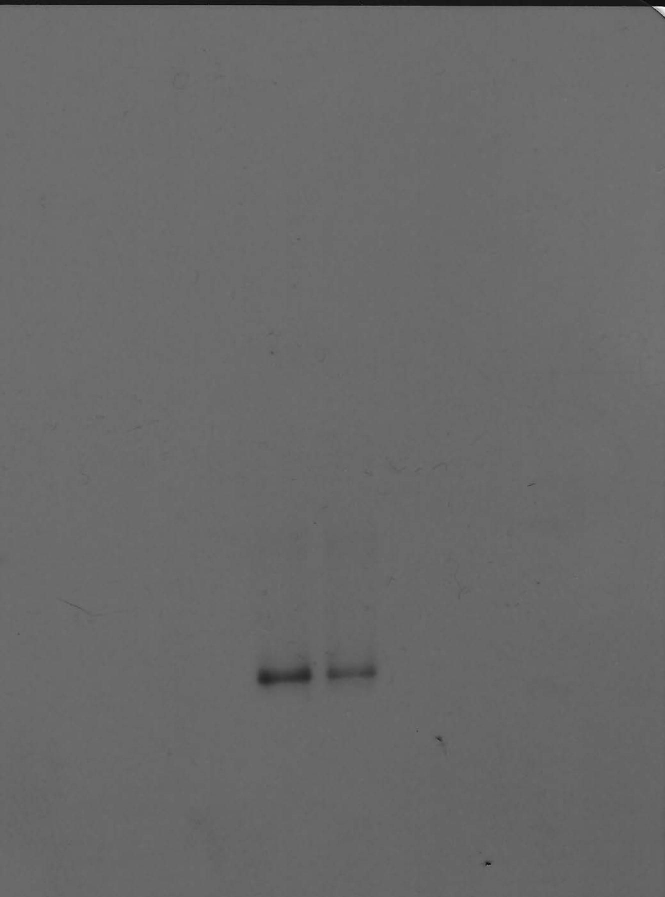

Figure 1C VPS50

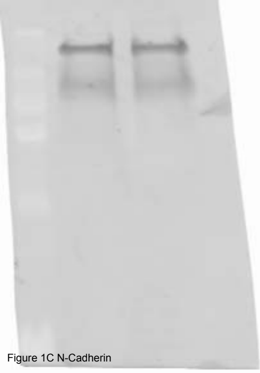

Figure 1C N-Cadherin

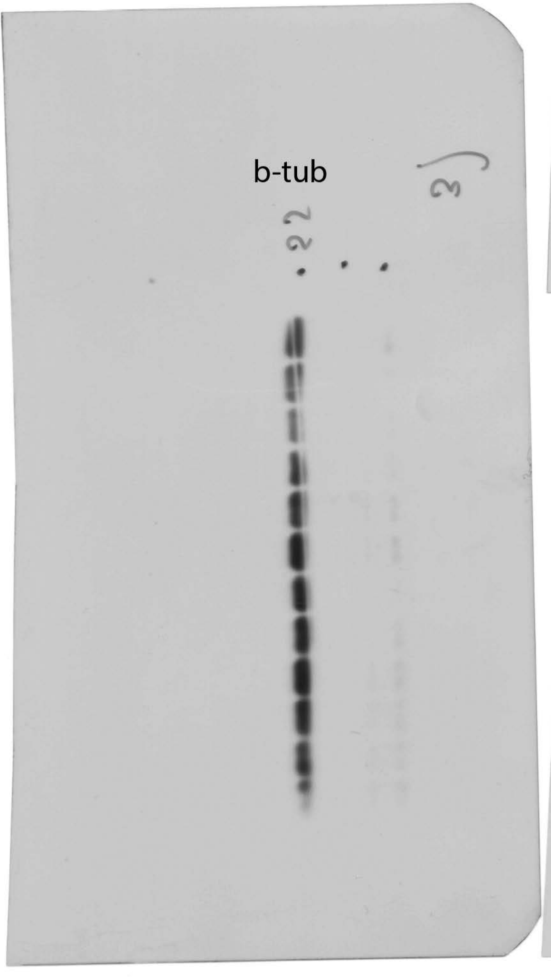

Figure 4D B-Tub

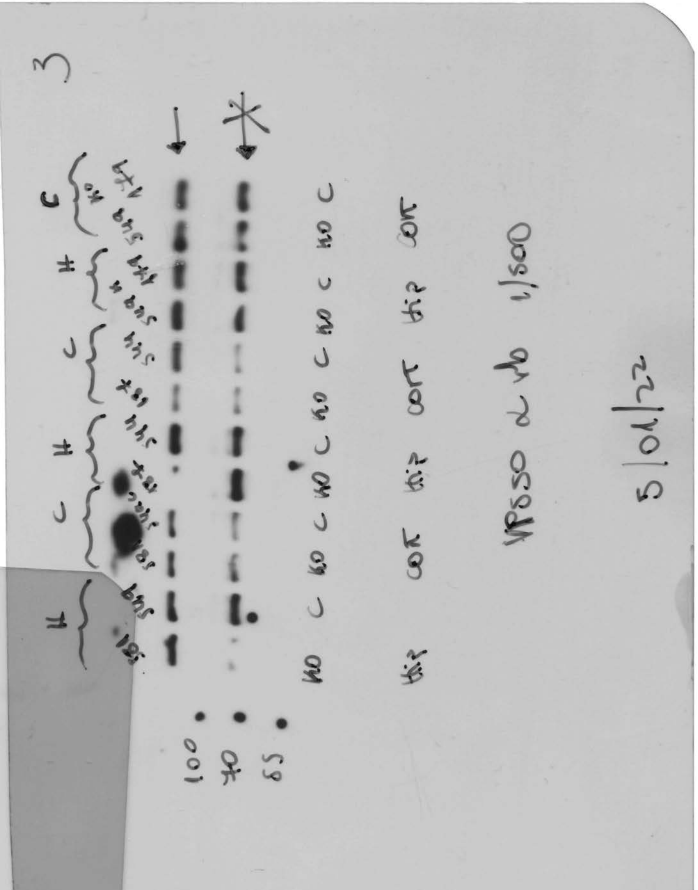

Figure 4D VPS50

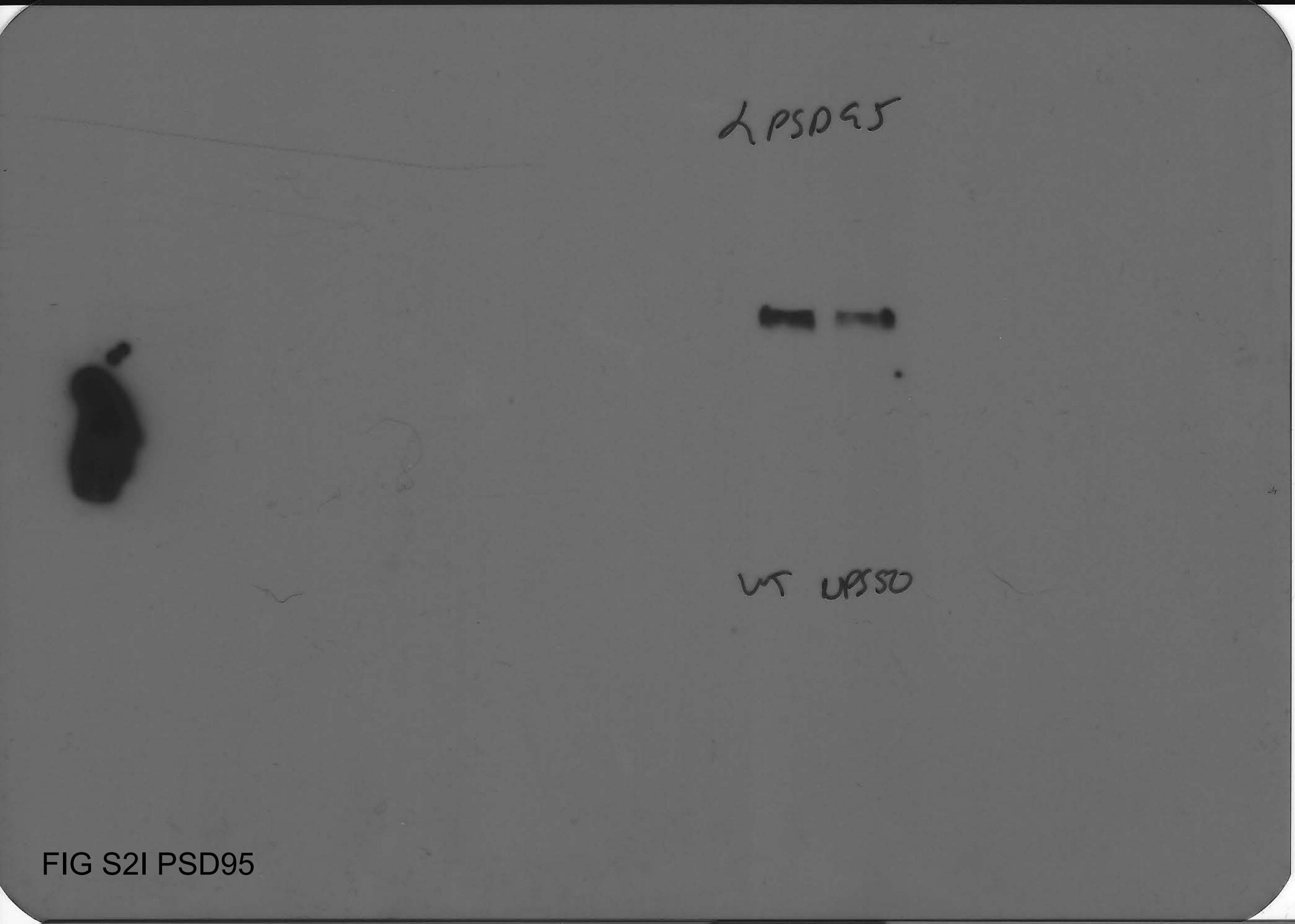

FIG S2I PSD95

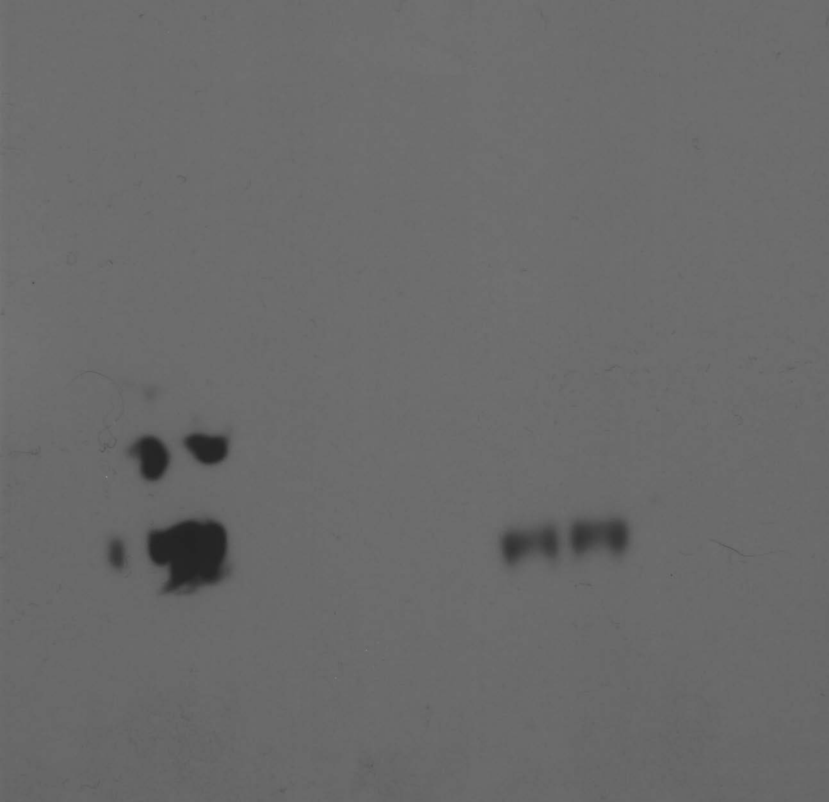

FIG S2I Syn

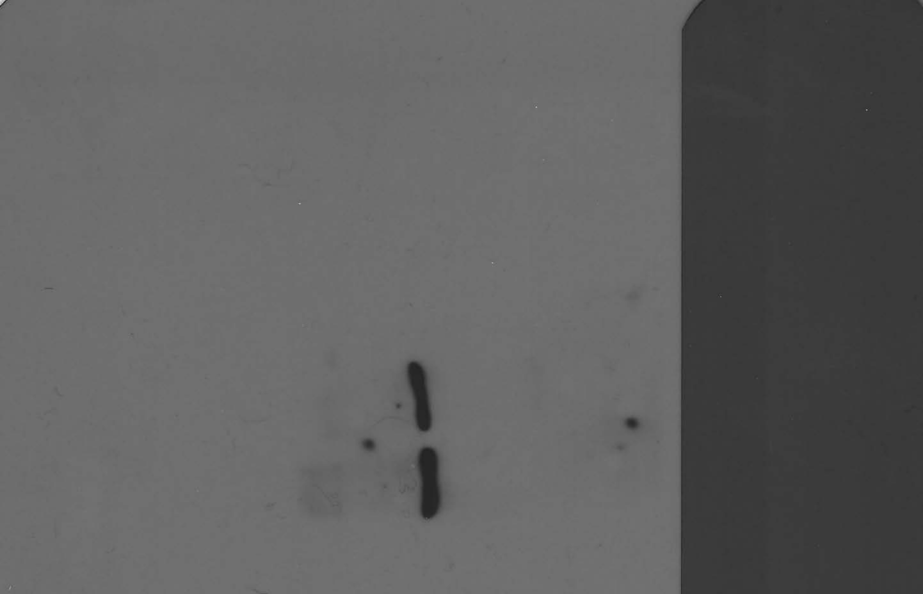

FIG. S2I N-Cadherin
